# Evaluation of the efficiency of entomopathogenic nematodes exposed to different temperatures of vinasse in the control of *Stomoxys calcitrans* (Linnaeus, 1758) (Diptera: Muscidae)

**DOI:** 10.29374/2527-2179.bjvm003824

**Published:** 2024-08-14

**Authors:** Américo de Castro Monteiro, Graziele Calixto Souza, Ana Caroline Ferreira de Souza, Danielle Pereira da Silva, João Luiz Lopes Monteiro, Melissa Carvalho Machado do Couto Chambarelli, Avelino José Bittencourt

**Affiliations:** 1 Veterinarian, DSc. Departamento de Parasitologia Animal (DPA). Instituto de Veterinária, Universidade Federal Rural do Rio de Janeiro. Seropédica, RJ. Brazil.; 2 Veterinarian, MSc. Autonomus. Rio de Janeiro, RJ. Brazil.; 3 Veterinarian, MSc. Programa de Pós-Graduação em Ciências Veterinárias (PPGCV), DPA. IV, UFRRJ. Seropédica, RJ. Brazil.; 4 Agronomist, DSc. Programa de Pós-Graduação em Agronomia, Departamento de Engenharia Agrícola. Centro de Ciências Agrárias, Universidade Federal de Roraima. Cauamé, RR. Brazil.; 5 Veterinarian, DSc. PPGCV, DPA. IV, UFRRJ. Seropédica, RJ. Brazil.; 6 Veterinarian, DSc. Departamento de Medicina e Cirurgia Veterinária (DMCV), IV, UFRRJ. Seropédica, RJ, Brazil.

**Keywords:** sugarcane, stable fly, biological control, vinasse, cana-de-açúcar, mosca dos estábulos, controle biológico, vinhaça

## Abstract

The sugarcane industry generates byproducts that contribute to the proliferation of *Stomoxys calcitrans*. An analysis was carried out to verify the efficacy of *Heterorhabditis bacteriophora* HP88 and *H. baujardi* LPP7 at different vinasse temperatures to control *S. calcitrans* larvae. Ten fly larvae were deposited in plastic containers containing four mL of 50% vinasse. Each treatment consisted of 300 EPN/larvae of *H. bacteriophora* added to the containers and heated at temperatures of 25, 28, 31, 34, 37 and 40 °C. The same treatments were performed using *H. baujardi*. The treatments were carried out in a BOD incubator at 25 ± 1 °C and 70 ± 10% RH, and each treatment was replicated six times. The treated groups, controls and temperatures showed no statistical differences in terms of larval mortality rate (P=0.8573), percentage of dead pupae (P=0.1782) and adult emergence (P=0.4386). Larval mortality rates of 30% and 14.17% were achieved with *H. bacteriophora* and *H. baujardi*, respectively, while the control groups presented 3.89% with *H. bacteriophora* and 8.61% with *H. baujardi*. From the standpoint of temperatures, significant differences were found only at 37 and 40 °C for *H. baujardi*. The highest pupal mortality achieved with *H. bacteriophora* was 34.17% at 31 °C, while that reached with *H. baujardi* at 37 °C was 40%. The groups containing *H. bacteriophora* caused lower adult emergence rates at temperatures of 25, 28, 31 and 34 °C, while *H. baujardi* caused its lowest emergence rates at 37 and 40 °C. It is concluded that infection occurs in the immature stages of *S. calcitrans* by EPN when added to 50% vinasse solution at different temperatures and that nematodes caused negative effects on the emergence of fly larvae at varying temperatures.

## Resumo

A indústria da cana-de-açúcar gera subprodutos que ajudam na proliferação de *Stomoxys calcitrans*. Uma análise foi realizada para verificar a eficiência de *Heterorhabditis bacteriophora* HP88 e *H. baujardi* LPP7 em diferentes temperaturas de vinhaça no controle de larvas de *S. calcitrans*. Dez larvas da mosca foram depositadas em recipientes plásticos contendo quatro mL de vinhoto à 50%. Em cada tratamento adicionou-se 300 NEP/larva de *H. bacteriophora*, aquecidos nas temperaturas de 25, 28, 31, 34, 37 e 40 °C. Os mesmos tratamentos foram realizados utilizando e *H*. *baujardi.* Os tratamentos foram realizados e mantidos em câmara climatizada a 25 ± 1 °C e 70 ± 10% UR, foram realizadas seis repetições para cada tratamento. Não houve diferença estatística entre os grupos tratados, controles e temperaturas para taxa de mortalidade de larval (*P*=0,8573), percentual de pupas mortas (*P*=0,1782) e emergência de adultos (*P*=0,4386). Foram observadas taxas de mortalidade larval de 30% e 14,17% para *H. bacteriophora* e *H*. *baujardi*, respectivamente, enquanto os grupos controles apresentaram 3,89% no *H. bacteriophora* e 8,61% *H*. *baujardi*. Na avaliação das temperaturas, foram observadas diferenças significativas apenas nas temperaturas 37 e 40 °C de *H. baujardi*. A maior mortalidade pupal observada para *H. bacteriophora* foi de 34,17% quando em 31 °C, já para *H*. *baujardi* na temperatura de 37 °C apresentou 40% de mortalidade. Houve menor emergência de adultos nas temperaturas de 25, 28, 31 e 34 °C nos grupos com *H. bacteriophora*, já *H*. *baujardi* causou as menores taxas de emergência quando em 37 e 40 °C. Conclui-se que ocorre infecção nos estágios imaturos de *S. calcitrans* por NEP quando adicionados à solução de vinhoto a 50% em diferentes temperaturas e que os nematoides causaram efeitos negativos na emergência da mosca em temperaturas variadas.

## Introduction

The growing sugarcane production in Brazil has generated byproducts, such as straw, sugarcane bagasse, filter cake, ash and vinasse, which enable the proliferation of the stable fly *Stomoxys calcitrans* (Linnaeus, 1758) (Dominghetti et al., 2015). This fly is a hematophagous dipteran parasite of several species of animals (Bishopp, 1913), including humans (Bittencourt, 2012).

The activity of this arthropod leads to estimated annual losses of 2,221 billion dollars (Taylor et al., 2012) in the United States, and 335,5 million dollars in Brazil (Grisi et al., 2014). However, these losses do not take into account the impact of recent outbreaks that have been reported in some regions of Brazil (Barros et al., 2010). *S. calcitrans* outbreaks have occurred recently, even during the colder months of the year, with an increase in dipterans between the months of April and May, which coincides with the sugarcane harvesting time (up to December) (Dominghetti et al., 2015).

The immature stages of the stable fly are favored by the large quantities of byproducts generated by sugar and alcohol mills, because during the sugarcane harvest, sugarcane fields are fertigated with vinasse, creating a favorable environment for stable fly outbreaks to occur (Dominghetti et al., 2015). As early as the 1980s, vinasse has been known to contribute to the development of *S. calcitrans* because it releases ammonia during its fermentation, attracting these dipterans and stimulating their egg laying (Buralli et al., 1987). In 2010, Barros et al. (2010) reported an outbreak of stable flies in the state of Mato Grosso do Sul, and the authors attributed the large number of flies to the irrigation of sugarcane fields with vinasse.

Due to pest resistance to chemical pesticides, alternatives are sought for the control of economically important parasites, through research into biological control and/or integrated control. These efforts have led to the proposal of several agents, including entomopathogenic nematodes (EPN), as alternatives for use in biological pest control (Alves, 1998).

EPNs have high virulence and specificity against arthropods; they are mobile, easily bred in vitro, and have remarkable reproductive potential. These characteristics favor the use of EPNs as pest control agents that have stages of development in the soil, since that is where these nematodes occur naturally (Kaya & Gaugler, 1993).

The purpose of this study was to evaluate the effect of entomopathogenic nematodes *Heterorhabditis bacteriophora* HP88 and *H. baujardi* LPP7 exposed to different vinasse temperatures on the mortality of *S. calcitrans* larvae, as well as on pupal mortality and adult emergence.

## Material and methods

The *S. calcitrans* colony used in this study was raised in a laboratory bench environment (27 ± 1 °C and 70-80% relative humidity – RH), using an adapted version of the method described by Macedo et al. (2005). The EPN colony was raised using the method described by Lindegren et al. (1993), i.e., maintained and multiplied in vivo on the moth *Galleria mellonella* (Lepidoptera: *Pyralidae*). The infective juveniles (IJs) were stored in an Eletrolab EL 202/4 B.O.D incubator at 16 ± 1 °C and 70-80% RH in a 40 mL cell culture flask for seven days. To calculate the dosages used in this study, the IJs were counted in twelve aliquots of 10 μL taken from an aqueous suspension of EPN. After counting the IJs in the 12 aliquots, the highest and lowest number of EPN/aliquot were discarded and the average number of IJs in the remaining 10 aliquots was calculated. Based on this calculation, the concentration of the suspensions was adjusted to IJs/mL (Taylor et al., 1998).

### Exposure of immature stages of *Stomoxys calcitrans* to nematodes

Ten third-instar larvae (9-10 days old)of stable fly were placed in plastic containers (7.5×7.5×4 cm), containing two sheets of filter paper, three grams of larval development diet and four mL of 50% vinasse solution. The pure vinasse, distilled water and nematode suspension were heated in a water bath for 10 minutes, at temperatures of 25, 28, 31, 34, 37 and 40 °C.

After heating, a 50% vinasse solution containing EPN was prepared. Each treatment consisted of 300 EPN/larva of *H. bacteriophora* HP88, contained in the above-described vinasse solution. The same procedure was repeated, but using *H. baujardi* LPP7. The control groups for each treatment received the same 50% vinasse solution heated in a water bath at the same temperatures as the treated groups. The treated and control groups were kept in an Eletrolab EL 212/4 BOD incubator at 25 ± 1 °C and 70 ± 10% RH, where larval mortality, pupal formation and adult emergence were observed daily for 15 days. Six replications were performed.

### Statistical analysis

The statistical design used here was completely randomized in a 2×2×6 factorial design (with and without the presence of nematodes, two types of EPN – *H. bacteriophora* and *H. baujardi* and at six temperatures) with six replications. Shapiro-Wilk and Kolmogorov-Smirnov normality tests were performed. Larval mortality rates, percent dead pupae and adult emergence were calculated using the GLIMMIX procedure. Interactions were broken down to 5% significance. The maximum significance level of 5% was used when differences between the means were detected. The analyses were carried out using SAS 9.4 software.

## Results

No correlation was found between the treated groups and control groups, entomopathogenic nematodes and temperatures for larval mortality rate (*P=*0.8573), percent dead pupae (*P=*0.1782) and adult emergence (*P=*0.4386).

The group treated with the nematode *H. bacteriophora* HP88 showed a significant difference, with the highest larval mortality rate detected in the group with the presence of EPN (30.00%), which was 87.0% higher than in the group without EPN (3.89%). No significant difference was found in the group treated with *H. baujardi* LPP7, which showed an average of 11.39% larval mortality rate. A comparison of the groups revealed a significant difference in the group with the presence of EPN, and the highest larval mortality rate was observed in the group treated with *H. bacteriophora* HP88 (30.00%), which was 52.8% higher than that achieved with *H. baujardi* LPP7 (14.17%) (Table 1).

**Table 1:** General average of larval mortality and of *Stomoxys calcitrans* flies emerged in the groups with and without the presence of the entomopathogenic nematodes *Heterorhabditis bacteriophora* –HP88 strain and *Heterorhabditis baujardi* – LP77 strain.

Group	EPN		*P*
	HP88	LPP7	
	Larval mortality (%)		
Presence of EPN	30.00 ± 3.47aA	14.17 ± 2.56aB	<.0001
Absence of EPN	3.89 ± 0.82bA	8.61 ± 2.68aA	
	Emergence of adults (%)		
Presence of EPN	38.05 ± 3.18bB	61.11 ± 5.05bA	<.0001
Absence of EPN	76.67 ± 2.58aA	75.00 ± 4.55aA	

EPN = entomopathogenic nematodes; *P* = probability; HP88 = *Heterorhabditis bacteriophora*; LPP7 = *Heterorhabditis baujardi*. Means followed by different lowercase letters in the columns and uppercase letters in the rows differ from each other by the Glimmix procedure.

In the assessment of adult emergence, groups with the presence of EPN showed lower adult emergence than groups without EPN. The lowest emergence percentage observed in the group with the presence of EPN was in the group treated with *H. bacteriophora* HP88 (38.05%). On the other hand, the group treated with *H. baujardi* LPP7 presented an emergence percentage of 61.11%, which was 37.7% higher than *H. bacteriophora* HP88. In the absence of EPN, the two groups showed no significant difference (Table 1).

As for larval mortality rate, the group containing *H. bacteriophora* HP88 showed no statistically significant difference, with an average mortality of 16.94%. However, there was a significant difference in the group containing *H. baujardi* LPP7, where the highest mortality rates occurred at temperatures of 37 and 40 °C, with an average of 26.25%, i.e., 84.9% higher than the other temperatures tested. When considering temperatures, differences were observed at 25, 28, 34 and 40 °C, and larval mortality rates were higher in the group containing *H. bacteriophora* HP88 than in the group with *H. baujardi* LPP7 (Table 2).

**Table 2:** Effect of the EPNs *Heterorhabditis bacteriophora* – HP88 strain and *Heterorhabditis baujardi* – LPP7 strain on *Stomoxys calcitrans* larvae exposed to different vinasse temperatures, kept in a B.O.D incubator at 25 ± 1 °C and 70-80% relative humidity.

Temperature (°C)	EPN		*P*
	HP88	LP77	
	Larval mortality (%)		
25	17.50 ± 5.52aA	4.17 ± 2.29bB	<.0001
28	15.83 ± 5.29aA	0.83 ± 0.83bB	
31	15.00 ± 6.57aA	5.83 ± 2.60bA	
34	22.50 ± 7.40aA	5.00 ± 2.61bB	
37	20.00 ± 6.40aA	20.83 ± 5.29aA	
40	10.83 ± 3.13aA	31.67 ± 4.58aB	
	Pupal mortality (%)		
25	32.50 ± 3.05aA	6.67 ± 3.10cB	<.0001
28	25.00 ± 3.79abA	10.00 ± 3.01cB	
31	34.17 ± 3.13aA	9.17 ± 3.36cB	
34	27.50 ± 3.28aA	20.00 ± 5.22bcB	
37	10.00 ± 5.36bB	40.00 ± 5.50aA	
40	22.50 ± 5.79abA	37.50 ± 6.17abA	
	Emergence of adults (%)		
25	50.00 ± 6.96aB	89.17 ± 3.98aA	<.0001
28	59.17 ± 5.96aB	89.17 ± 3.13aA	
31	50.83 ± 6.45aB	85.00 ± 3.99aA	
34	50.00 ± 7.88aB	75.00 ± 6.34aA	
37	70.00 ± 8.26aA	39.17 ± 5.70bB	
40	64.17 ± 8.57aA	30.83 ± 6.33bB	

EPN = entomopathogenic nematodes; *P* = probability; HP88 = *Heterorhabditis bacteriophora*; LPP7 = *Heterorhabditis baujardi*. Means followed by different lowercase letters in the columns and uppercase letters in the rows differ from each other by the Glimmix procedure.

Pupal mortality showed a statistically significant difference between the two EPN species evaluated. In the group containing *H. bacteriophora* HP88, pupal mortality was higher at temperatures of 25, 31 and 34 °C, with an average of 31.39% (Table 2). In the group with *H. baujardi* LPP7, pupal mortality rates were higher at temperatures of 37 °C and 40 °C, with an average mortality of 38.75%. Conversely, at lower temperatures, pupal mortality was observed at temperatures of 25, 28 and 31 °C, with an average mortality rate of 8.61% (Table 2).

The evaluation of adult emergence indicated no significant differences in the group with the EPN *H. bacteriophora* HP88, which showed an average of 57.36%. Conversely, *H. baujardi* LPP7 showed a significant difference in terms of adult emergence rates at temperatures of 25, 28, 31 and 34 °C, with an average of 84.58%, which was higher than the average of 35.00% reached at temperatures of 37 °C and 40 °C (Table 2). When considering temperatures, significant differences were observed at temperatures of 25, 28, 31 and 34 °C, in the group with *H. baujardi* LPP7, which showed a higher adult emergence rate. The highest adult emergence rates at temperatures of 37 and 40 °C were observed in the group with *H. bacteriophora* HP88 (Table 2).

## Discussion

The present study will bring to light several doubts regarding the behavior of nematodes against stable fly larvae at different vinasse temperatures, bringing field tests using these biological agents increasingly closer, because there are no similar studies with *S. calcitrans* and EPN in the same conditions.

Shapiro-Ilan et al. (2003), who evaluated the virulence of entomopathogenic nematodes (*H. bacteriophora, Steinernema carpocapsae, S. glaseri, and S. rarum*) and fungi collected from various locations in the USA, found that 30% of mortality in larvae of beetles from the family Curculionidae in peach plantations occurred due to the activity of these agents. The average mortality rate of *S. calcitrans* larvae achieved in the present study was also 30% in the group containing *Heterorhabditis bacteriophora* HP88.

Mendes et al. (2016) demonstrated emergence rates of *S. calcitrans* adults above 74% when the fly larvae were raised on a diet containing vinasse and sugarcane ash. In contrast, in the present study, the adult emergence rate decreased in the group with *H. bacteriophora* HP88, regardless of the temperatures used, indicating the activity of EPN, since the average emergence rate in this group was 57.36%.

The group containing *H. baujardi* LPP7 showed low adult emergence rates only at temperatures of 37 and 40 °C, i.e., 39.17% and 30.83%, respectively. This indicates that EPN continued to be active inside the fly larvae after infecting them. No significant difference in larval mortality rates was found in the groups with *H. bacteriophora* HP88 between the temperatures, even though higher averages were observed in the groups subjected to 34 and 37 °C. However, the group containing *H. baujardi* LPP7 showed a higher larval mortality at relatively higher temperatures (37 and 40 °C), proving to be more efficient at these temperatures. This finding differs from the results reported by Kaya (1990), who found that the best temperatures for EPN (*H. bacteriophora*, S. *carpocapsae* and *S. glaseri*) activity in the soil were 12 to 28 °C. On the other hand, Minas et al. (2011) reported a higher larval mortality rate of the Mediterranean fly (*Ceratitis capitata*) at a temperature of 28 °C with *H. baujardi* LPP7.

Leal et al. (2017) when using *H. bacteriophora* HP88 and *H. baujardi* LPP7, attributed pupal mortality to infection during the larval period, preventing the formation of the adult insect inside the pupae by damaging the contents of the pupae. This activity in pupae was also observed in both groups containing EPN in the present study. Minas et al. (2011) reported pupal mortality and decreased viability of *Ceratitis capitata* after exposure to *H. baujardi* LPP7, with pupal mortality exceeding 90% at 24 and 28 °C. This mortality is related to the concentration of EPN, as Minas et al. (2011) demonstrated a direct relationship between EPN concentration and mortality, unlike the findings of our study, in which only the concentration of 300 EPN/larva was evaluated.

Lysyk (1998) reported high survival rates of immature stages of *S. calcitrans* at temperatures of 20-23 °C. In the present study, a reduction in the emergence of adults was observed in groups with the presence of EPN. This reduction was observed at all the temperatures applied to the group containing *H. bacteriophora* HP88, but was observed only at temperatures 37 and 40 °C in the group containing *H. baujardi* LPP7.

Mukuka et al. (2010) analyzed natural populations of *H. bacteriophora* HP88 exposed to high temperatures, and identified a tolerance range of 33.3 °C to 40.1 °C, pointing out that higher temperatures can affect the growth, infectivity and reproduction of EPN. In the present study, the infectivity and reproduction of EPN in larvae was found to decrease in the group with *H. bacteriophora* HP88 exposed to 40 °C, which reached a larval mortality rate of 10.83%.

Monteiro Sobrinho et al. (2016) demonstrated that larval mortality increases as the concentrations of EPN/*S. calcitrans* larvae in filter cake increase. However, Mahmoud et al. (2007), who used *Steinernema feltiae* to control *S. calcitrans* larvae, observed 100% larval mortality at concentrations of 80 and 100 EPN/larva, which is superior to the results reported by Monteiro Sobrinho et al. (2016). However, the study by Mahmoud et al. (2007) did not use filter cake substrate, which may also have interfered with the activity of EPN. Therefore, the same thing may have occurred in the present study, where there was the presence of EPN together with high concentrations of vinasse. This fact apparently may also have caused a drop in the efficacy of the EPN.

Monteiro-Sobrinho et al. (2023) reported that when the EPN *H. bacteriophora* HP88 and *H. baujardi* LPP7 were subjected to a 50% concentration of vinasse at 35 °C, they were able to kill 100 and 70%, respectively, of the stable fly larvae. These values ​​are considerably higher than those found in the present study at all temperatures tested. This difference may be linked to the age of the fly larvae, since the aforementioned authors used 6-day-old larvae, while in the present study the fly larvae were between nine and 10 days old, considerably more resistant to the action of external agents.

The concentration of EPN in the work of Monteiro-Sobrinho et al. (2023) was 400 EPN/larva, while the present study used 300 EPN/larva. This difference in nematode concentrations may also have influenced the mortality caused to fly larvae.

Understanding the behavior of EPN against stable fly larvae at different concentrations of vinasse and temperatures is crucial for the future development of successful strategies for controlling this dipteran, which causes diverse health, economic and social impacts in Brazil.

## Conclusions

It is concluded that infection occurs in the immature stages of *S. calcitrans* by EPN when added to 50% vinasse solution at different temperatures and that nematodes caused negative effects on the emergence of fly larvae at varying temperatures.

The EPN showed promise in controlling immature stages of stable fly under the conditions presented in the present work.

## References

[B001] Alves S. B. (1998). Controle microbiano de insetos.

[B002] Barros A. T. M., Koller W. W., Catto J. B., Soares C. O. (2010). Surtos por *Stomoxys calcitrans* em gado de corte no Mato Grosso do Sul. Pesquisa Veterinária Brasileira.

[B003] Bishopp F. C. (1913). The stable fly (*Stomoxys calcitrans*: L.): an important livestock pest. Journal of Economic Entomology.

[B004] Bittencourt A. J. (2012). Avaliação de surtos e medidas de controle ambiental de *Stomoxys calcitrans* (Diptera: Muscidae) na Região Sudeste do Brasil. Revista Brasileira de Medicina Veterinária.

[B005] Buralli G. M., Born R. H., Gerola O., Pimont M. P. (1987). Soil disposal of residues and the proliferation of flies in the State of São Paulo. Water Science and Technology.

[B006] Dominghetti T. F. S., Barros A. T. M., Soares C. O., Cançado P. H. D. (2015). *Stomoxys calcitrans* (Diptera: Muscidae) outbreaks: Current situation and future outlook with emphasis on Brazil. Revista Brasileira de Parasitologia Veterinária.

[B007] Grisi L., Leite R. C., Martins J. R., Barros A. T. M., Andreotti R., Cançado P. H. D., Léon A. A. P., Pereira J. P., Villela H. S. (2014). Reassessment of the potential economic impact of cattle parasites in Brazil. Revista Brasileira de Parasitologia Veterinária.

[B008] Kaya H. K., Gaugler R. (1993). Entomopathogenic nematodes. Annual Review of Entomology.

[B009] Kaya H. K., Gaugler R., Kaya H. K. (1990). Entomopathogenic nematodes in biological control.

[B010] Leal L. C. S. R., Monteiro C. M. O., Mendonça A. E., Bittencourt V. R. E. P., Bittencourt A. J. (2017). Potential of entomopathogenic nematodes of the genus *Heterorhabditis* for the control of *Stomoxys calcitrans* (Diptera: Muscidae). Revista Brasileira de Parasitologia Veterinária.

[B011] Lindegren J. E., Valero K. A., Mackey B. E. (1993). Simple in vivo production and storage methods for *Steinernema carpocapsae* infective juveniles. Journal of Nematology.

[B012] Lysyk T. J. (1998). Relationship between temperature and life-history parameters of *Stomoxys calcitrans* (Diptera: Muscidae). Journal of Medical Entomology.

[B013] Macedo D. M., Chaaban A., Moya-Borja G. E. (2005). Desenvolvimento pós-embrionário de *Stomoxys calcitrans* (Linnaeus, 1758) (Diptera: Muscidae) criadas em fezes de bovinos tratados com diferentes avermectinas. Revista Brasileira de Parasitologia Veterinária.

[B014] Mahmoud M. F., Mandour N. S., Pomazkov I. Y. (2007). Efficacy of the entomopathogenic nematode *Steinernema feltiae* Cross N 33 against larvae and pupae of four fly species in the laboratory. Nematologia Mediterranea.

[B015] Mendes C. D. O. F., Silva A. C., Leal L. C. D. S. R., Barbosa C. G., Bittencourt A. J. (2016). Biologia de *Stomoxys calcitrans* (Diptera: Muscidae) em subprodutos da indústria sucroalcooleira. Revista Brasileira de Medicina Veterinária.

[B016] Minas R. S., Dolinski C., Carvalho R. S., Souza R. M. (2011). Controle biológico da mosca-do-mediterrâneo *Ceratitis capitata* utilizando nematoides entomopatogênicos em laboratório. Scientia Agraria.

[B017] Monteiro A. C., Leal L. C. S. R., Monteiro J. L. L., Chambarelli M. C. M. C., Bittencourt A. J. (2023). Evaluation *in vitro* of the virulence of two entomopathogenic heterorhabditid nematodes in the control of *Stomoxys calcitrans* (Diptera: Muscidae) larvae in byproducts of the sugar and alcohol industry. Revista Brasileira de Parasitologia Veterinária.

[B018] Monteiro A. C., Mendes C. O. F., Leal L. C. S. R., Bittencourt A. J. (2016). Virulência de *Heterorhabditis bacteriophora* cepa HP88 (Rhabditida: Heterorhabtidae) sobre larvas de *Stomoxys calcitrans* (Díptera: Muscidae) em dieta de torta de filtro. Revista Brasileira de Medicina Veterinária.

[B019] Mukuka J., Strauch O., Hoppe C., Ehlers R. U. (2010). Improvement of heat and desiccation tolerance in *Heterorhabditis bacteriophora* through cross breeding of tolerant strains and successive genetic selection. BioControl.

[B020] Shapiro-Ilan D. I., Gardner W. A., Fuxa J. R., Wood B. W., Nguyen K. B., Adams B. J., Hall M. J. (2003). Survey of entomopathogenic nematodes and fungiendemic to pecan orchards of the Southeastern United States and their virulence to the pecan weevil (Coleoptera: Curculionidae). Environmental Entomology.

[B021] Taylor D. B., Moon R. D., Mark D. R. (2012). Economic impact of stable flies (Diptera: Muscidae) on dairy and beef cattle production. Journal of Medical Entomology.

[B022] Taylor D. B., Szalanski A. L., Adams B. J., Peterson II R. D. (1998). Susceptibility of house fly (Diptera: Muscidae) larvae to entomopathogenic nematodes (Rhabditida: Heterorhabditidae, Steinernematidae). Environmental Entomology.

